# FIGO good practice recommendations on anemia in pregnancy, to reduce the incidence and impact of postpartum hemorrhage (PPH)

**DOI:** 10.1002/ijgo.70529

**Published:** 2025-10-01

**Authors:** Akaninyene E. Ubom, Ferdousi Begum, Diana Ramasauskaite, Albaro J. Nieto‐Calvache, Monica Oguttu, Inês Nunes, Zechariah J. Malel, Jolly Beyeza‐Kashesya, Alison Wright, the FIGO Committee on Childbirth and Postpartum Hemorrhage

**Affiliations:** ^1^ Department of Obstetrics and Gynecology, Faculty of Clinical Sciences PAMO University of Medical Sciences Port Harcourt Nigeria; ^2^ World Association of Trainees in Obstetrics & Gynecology (WATOG) Paris France; ^3^ Department of Obstetrics & Gynecology Institute of Woman and Child Health Dhaka Bangladesh; ^4^ Center of Obstetrics and Gynecology Vilnius University Medical Faculty Vilnius Lithuania; ^5^ Departamento de Ginecología y Obstetricia Fundación Valle del Lili Cali Colombia; ^6^ Faculty of Health Sciences Universidad ICESI Cali Colombia; ^7^ Kisumu Medical and Education Trust Kisumu Kenya; ^8^ Department of Obstetrics and Gynecology Gaia/Espinho Local Health Unit Porto Portugal; ^9^ CINTESIS—Center for Health Technology and Services Research University of Porto Porto Portugal; ^10^ Department of Medical Sciences University of Aveiro Aveiro Portugal; ^11^ Department of Obstetrics and Gynecology, School of Medicine University of Juba South Sudan; ^12^ Association of Gynecologists and Obstetricians of South Sudan (AGOSS) Juba South Sudan; ^13^ Mulago Specialised Women and Neonatal Hospital Kampala Uganda; ^14^ Royal Free London NHS Foundation Trust London UK; ^15^ University College London London UK

**Keywords:** folic acid, hemoglobinopathy, iron, malaria, maternal anemia, multiple micronutrient

## Abstract

Anemia affects 32 million pregnant women globally, contributing annually to more than 115 000 maternal deaths and 591 000 perinatal deaths worldwide. Low‐ and middle‐income countries (LMICs) bear the highest burden of anemia in pregnancy, with nearly 50% of affected pregnant women. It is now 2025, which is WHO's target year for a 50% reduction in maternal anemia, and the global prevalence of anemia in pregnancy remains more than twice the target of 15%. This calls for a renewed global focus on optimal approaches for reducing the burden and complications of anemia in pregnancy. In this FIGO Childbirth and Postpartum Hemorrhage (PPH) Committee paper, current best evidence on anemia in pregnancy has been reviewed and synthesized, to make recommendations on screening, diagnosis, prevention, and treatment of anemia in pregnancy. We recommend that all pregnant women should be screened for anemia in pregnancy at booking and again at 28 weeks of pregnancy, with a full blood count (FBC), or packed cell volume/hemoglobin concentration in settings where FBC is not available. A hemoglobin concentration cutoff of less than 11 g/dL in all trimesters of pregnancy and during the postpartum period, as well as in all settings and populations, is recommended for the diagnosis of anemia in pregnancy. Routine iron and folic acid supplementation, either alone, or as components of multiple micronutrient supplements, is also recommended during pregnancy. We also made recommendations for malaria and anti‐helminthic chemoprophylaxis, hemoglobinopathy screening, iron, folate, and multiple micronutrient supplementation, and blood transfusion in pregnant women with hemoglobinopathies. Finally, the relationship between anemia and postpartum hemorrhage is highlighted.

## 1 SUMMARY OF FIGO RECOMMENDATIONS ON THE SCREENING, DIAGNOSIS, AND PREVENTION OF ANEMIA IN PREGNANCY

### 1.1 Screening and diagnosis of anemia in pregnancy

1. All pregnant women should be screened for anemia with a full blood count (FBC) at booking and again at 28 weeks (strong, low). In resource‐limited settings, a hemoglobin concentration or hematocrit test can be used for screening (conditional, low).
2. Anemia in pregnancy is diagnosed if the hemoglobin concentration is less than 11.0 g/dL in all trimesters of pregnancy and during the postpartum period (strong, low).
3. A hemoglobin concentration cutoff of less than 11 g/dL should be universally adopted in all settings and populations for the diagnosis of anemia in pregnancy (strong, low).
4. Anemia in pregnancy is classified based on severity as mild (10–10.9 g/dL), moderate (7.0–9.9 g/dL), and severe (<7.0 g/dL) (conditional, low).

### 1.2 Iron supplementation and prevention of iron deficiency anemia in pregnancy

5 To prevent iron deficiency anemia, all pregnant women should receive a daily oral iron supplementation of 30–60 mg of elemental iron (strong, high). A higher daily dose of 60 mg is recommended in settings where iron deficiency anemia is a significant public health problem affecting 40% or more of pregnant women, and in women at increased risk of iron deficiency, including women with previous anemia, multiple pregnancy, short interpregnancy interval of less than 1 year, and vegetarians (conditional, low).
6 Women who do not tolerate daily oral iron due to side effects should receive intermittent supplementation with 120 mg of elemental iron weekly (strong, high). Intermittent supplementation can also be considered in settings where iron deficiency anemia affects less than 20% of the pregnant population (conditional, low).
7 To maximize absorption, women should be advised to take oral iron in the morning on an empty stomach (1 h before eating or 2 h after), and 1 h before or 2 h after inhibitors of iron absorption such as calcium, proton pump inhibitors, antacids, thyroxine, tea, coffee, milk, soy, and eggs (conditional, low).
8 Administration of vitamin C with oral iron to enhance iron absorption can be considered (conditional, low). Evidence for this is conflicting and more studies are needed to make definite recommendations.

### 1.3 Treatment of iron deficiency anemia in pregnancy

9 Oral iron is recommended as the first‐line treatment for iron deficiency anemia at a dose of 60–120 mg of elemental iron daily (strong, low). This should be continued until hemoglobin concentration normalizes at 11.0 g/dL or above, after which the dose should be reduced to the prophylactic dose of 30–60 mg of elemental iron daily and continued for 3 months or 6 weeks postpartum, whichever is longer, to replenish iron stores (strong, low).
10 Parenteral iron is indicated for the treatment of iron deficiency anemia in women who do not respond, are non‐compliant, or are intolerant to oral iron, moderate to severe iron deficiency anemia, iron deficiency anemia occurring within 4–6 weeks of anticipated childbirth, and women with a history of gastric surgery or conditions such as inflammatory bowel disease, which impair absorption of oral iron (strong, high). Parenteral iron is not recommended in the first trimester owing to paucity of safety data (conditional, low).
11 Blood transfusion is indicated in women with severe anemia, especially when it is close to the due date of childbirth, acute severe bleeding or where there is a risk of further bleeding, and for significant symptomatic anemia with features of hemodynamic or cardiac compromise (strong, low).

### 1.4 Folic acid supplementation in pregnancy and pre‐pregnancy

12 Daily oral folic acid supplementation with 400 μg (0.4 mg) of folic acid (given together with oral iron) is recommended for all pregnant women to prevent maternal anemia and other complications of folate deficiency in pregnancy (strong, high). Taking folic acid on an empty stomach provides the highest bioavailability (conditional, low).
13 Intermittent oral folic acid supplementation with 2800 μg (2.8 mg) of folic acid weekly (given together with iron) is also effective for the prevention of maternal anemia and other complications of folate deficiency in pregnancy (strong, high), and should be considered in women who do not tolerate the gastric side effects of daily oral iron/folic acid and in settings with an anemia prevalence in pregnancy of less than 20% (conditional, low).
14 For the prevention of fetal neural tube defects, daily oral folic acid supplementation with 400 μg of folic acid is recommended for all women of childbearing age, beginning at least 2–3 months before pregnancy and continued until 12 weeks of pregnancy (strong, high). This dose should be continued (together with iron) beyond 12 weeks and throughout pregnancy to prevent maternal anemia and other complications of folate deficiency in pregnancy aside from fetal neural tube defects (strong, high).
15 High daily dose of folic acid of 5 mg, commenced at least 2–3 months before pregnancy and continued until 12 weeks of pregnancy, is recommended for the prevention of fetal neural tube defects in women considered to be at high risk (strong, low). After 12 weeks of gestation, the dose should be reduced to 400 μg/day and taken (together with iron) throughout pregnancy to prevent maternal anemia and other complications of folate deficiency in pregnancy (strong, high).
16 Women with hemoglobinopathies, a body mass index (BMI, calculated as weight in kilograms divided by the square of height in meters) of 30 or above, and those on anti‐folate drugs should receive high dose folic acid of 5 mg/day from 2 to 3 months before pregnancy and throughout pregnancy for the prevention of fetal neural tube defects, maternal anemia, and other complications of folate deficiency in pregnancy (conditional, low).
17 Intermittent folic acid supplementation with 2800 μg (2.8 mg) of folic acid/week commenced at least 2–3 months before pregnancy and continued until 12 weeks of gestation is also effective for the prevention of fetal neural tube defects (strong, high).
18 Intermittent folic acid supplementation for the prevention of fetal neural tube defects, maternal anemia, and other complications of folate deficiency in pregnancy is not recommended for women with hemoglobinopathies, a BMI of 30 or above and on anti‐folate drugs, as these women require a high daily dose folic acid of 5 mg/day (conditional, low).

### 1.5 Vitamin B12 supplementation in pregnancy

19 While vitamin B12 supplementation during pregnancy may reduce the risks of maternal vitamin B12 deficiency and anemia, there is not enough evidence to recommend routine vitamin B12 supplementation in pregnancy (conditional, low).

### 1.6 Multiple micronutrient supplementation in pregnancy

20 Multiple micronutrient supplements (containing iron and folic acid) should be considered over iron and folic acid supplements alone for pregnant women in low‐ and middle‐income countries (LMICs) and in populations with a high prevalence of nutritional deficiencies among women of reproductive age (strong, high).
21 Multiple micronutrient supplements used in pregnancy should contain the following 15 micronutrients at doses that meet the recommended dietary allowances for these micronutrients: folic acid, iron, copper, iodine, selenium, vitamin A, vitamin B1, vitamin B2, vitamin B3, vitamin B6, vitamin B12, vitamin C, vitamin D, vitamin E, and zinc (strong, high).
22 Multiple micronutrient supplements should be administered once daily throughout pregnancy (strong, high). For the prevention of fetal neural tube defects, they should be started at least 2–3 months before pregnancy (strong, high).
23 Intermittent/weekly administration of multiple micronutrient supplements in pregnancy is not recommended (conditional, low).
24 For women at low risk for fetal neural tube defects, multiple micronutrient supplements containing 400 μg of folic acid should be started 2–3 months before pregnancy, given once daily, and continued until 12 weeks of pregnancy (strong, high). This dose should be continued beyond 12 weeks and throughout pregnancy (strong, high).
25 For women at high risk of fetal neural tube defects, multiple micronutrient supplements containing 5 mg of folic acid should be started 2–3 months before pregnancy, given once daily, and continued until 12 weeks of pregnancy, and thereafter changed to multiple micronutrient supplements containing 400 μg of folic acid, which should be continued once daily throughout pregnancy (strong, high). Women with a BMI of 30 or above and on antifolate drugs, should maintain a high daily dose of 5 mg of folic acid throughout pregnancy (conditional, low). In the absence of a multiple micronutrient supplement containing 5 mg of folic acid, additional tablet(s) of folic acid should be taken in addition to the multiple micronutrient supplement to achieve a daily dose of 5 mg of folic acid (conditional, low).
26 It is not recommended for pregnant women to use multiple micronutrient supplements containing more than 30 mg of iron, owing to side effects (conditional, low). In situations where higher doses of 60–120 mg of iron are required, rather than use multiple micronutrient supplements containing such a high dose of iron, additional iron tablets should be administered in order to achieve the desired daily iron dose (conditional, low).

### 1.7 Malaria chemoprophylaxis in pregnancy

27 Intermittent preventive treatment with sulfadoxine‐pyrimethamine (IPT_P_‐SP) is recommended for all pregnant women in malaria‐endemic areas. IPT should be started as early as possible in the second trimester, with subsequent doses given at least 4 weeks apart until birth, ensuring at least three doses before birth (strong, high).
28 IPT_P_‐SP should be administered as directly observed therapy (DOT) in the antenatal clinic with three tablets of SP containing 1500 mg of sulfadoxine/75 mg of pyrimethamine (conditional, low).
29 High‐dose folic acid (≥5 mg) should not be co‐administered with SP, as it counteracts the efficacy of SP (strong, high).
30 In women on high‐dose folic acid, switching to low‐dose (400 μg–1.5 mg) folic acid for 14 days after administration of SP for IPT should be considered (conditional, low).
31 SP is contraindicated as follows: (1) before 13 weeks of pregnancy; (2) in women taking other sulfa‐containing drugs for prophylaxis; and (3) in women who are allergic to any of the components of SP (strong, low).
32 For pregnant women who are allergic to any of the components of SP, there is limited evidence to recommend alternative antimalarials for IPT (conditional, low). These women should be encouraged to use long‐lasting insecticidal nets and adopt other malaria prevention strategies, with early diagnosis and prompt treatment of clinical malaria (strong, high).

### 1.8 Anti‐helminthic prophylaxis in pregnancy

33 Preventive chemotherapy with a single‐dose of 400 mg albendazole or 500 mg mebendazole administered after the first trimester is recommended for all pregnant women residing in areas where both the prevalence of helminthiasis is 20% or greater and anemia is a severe public health issue affecting at least 40% of pregnant women (conditional, low).

### 1.9 Screening for hemoglobinopathy in pregnancy

34 All pregnant women should be screened with a FBC and hemoglobin electrophoresis at booking to detect hemoglobinopathies in pregnancy (strong, low).

### 1.10 Folic acid supplementation in pregnant women with hemoglobinopathy

35 Women with hemoglobinopathy should have folic acid supplementation with 5 mg/day of folic acid 2–3 months before and during pregnancy (strong, low). Folic acid supplementation can be provided with either a 5 mg folic acid tablet or a multiple micronutrient supplement containing 5 mg of folic acid (conditional, low). In the absence of a multiple micronutrient supplement containing 5 mg of folic acid, additional folic acid tablet(s) should be given to achieve a daily dose of 5 mg of folic acid (conditional, low).
36 Intermittent folic acid supplementation is not recommended in women with sickle cell disease as these women require a higher daily dose of 5 mg folic acid compared to the 2.8 mg weekly dose in intermittent supplementation (conditional, low).

### 1.11 Iron supplementation in pregnant women with hemoglobinopathy

37 Women with hemoglobinopathy should be given iron supplementation only if there is laboratory evidence of iron deficiency (i.e. ferritin <30 μg/L) (conditional, low).
38 In settings where a serum ferritin test is not readily available, iron supplementation at a dose of 30 mg of elemental iron/day can be safely administered to pregnant women with sickle cell disease who do not have a history of repeated blood transfusions (conditional, low). Iron supplementation can be provided with either iron tablet or a multiple micronutrient supplement containing iron (conditional, low). Iron supplementation is contraindicated with repeated blood transfusions (conditional, low).
39 Women who do not tolerate daily iron due to side effects can have intermittent supplementation with 120 mg of iron/week (strong, high).

### 1.12 Multiple micronutrient supplementation in pregnant women with hemoglobinopathy

40 Multiple micronutrient supplementation is beneficial and should be considered in pregnant women with sickle cell disease because of a higher risk of malnutrition and micronutrient deficiencies (conditional, low).

### 1.13 Blood transfusion in pregnant women with hemoglobinopathy

41 Blood transfusion is indicated to treat acute anemia in pregnant women with sickle cell disease if the hemoglobin concentration is less than 5–7 g/dL, or greater than 2 g/dL below the steady state hemoglobin concentration, or a reduction of more than 20% from the steady state hemoglobin concentration (conditional, low).
42 Routine prophylactic blood transfusion in pregnant women with sickle cell disease is not recommended (conditional, low).
43 Hemoglobin concentration in pregnant women with thalassemia syndromes should be maintained at a target of 10 g/dL with transfusions to avoid maternal and fetal morbidity (conditional, low).

### 1.14 Malaria chemoprophylaxis in pregnant women with hemoglobinopathy

44 Malaria chemoprophylaxis with daily proguanil is recommend for all pregnant women with sickle cell disease who reside in malaria‐endemic areas (strong, high).

### 1.15 Anemia and postpartum hemorrhage (PPH)

45 Universal adoption of a hemoglobin concentration of less than 11.0 g/dL for the diagnosis of anemia in pregnancy is recommended to reduce the risk of PPH (strong, low). Women who start pregnancy with a hemoglobin concentration of 11 g/dL or above can tolerate more postpartum blood loss, with a lower risk of PPH, than women with a hemoglobin concentration of 10 g/dL, which is the cutoff for diagnosing anemia in some African settings.
46 Prevention, screening, and correction of antepartum anemia is an important and effective strategy for reducing the risk of PPH (strong, high).

## 2 GRADING OF EVIDENCE AND STRENGTH OF RECOMMENDATIONS

The strength of recommendations and quality of evidence for the foregoing recommendations were rated using the Grading of Recommendations Assessment, Development and Evaluation (GRADE, 2011) approach. Recommendations for which we are confident that the benefits/desirable effects outweigh the risks/undesirable effects or that the risks/undesirable effects outweigh the benefits/desirable effects were rated as strong (strong recommendation for and against an intervention, respectively). A strong recommendation implies that most patients will benefit from the recommended intervention, and the quality of evidence is higher.

Recommendations for which the benefits/desirable effects probably outweigh the risks/undesirable effects or the risks/undesirable effects probably outweigh the benefits/desirable effects were rated as conditional (conditional recommendation for and against an intervention, respectively). Conditional recommendations are those for which the balance between benefits/desirable effects and risks/undesirable effects is small/uncertain, and the quality of evidence is lower. For interventions that are conditionally recommended, different choices/options will be appropriate for different categories of patients, and maternity staff should support each patient in reaching a management decision that is both beneficial and consistent with the patient's values and preferences.

For recommendations that are based on evidence from randomized controlled trials (RCTs) and systematic reviews and meta‐analyses (Cochrane and non‐Cochrane) of RCTs, the quality of evidence was rated as high, while evidence from observational studies (cross‐sectional, cohort, and case–control studies) or systematic reviews and meta‐analyses (Cochrane and non‐Cochrane) of observational studies were rated as low quality. Quality of evidence from RCTs or systematic reviews and meta‐analyses (Cochrane and non‐Cochrane) of RCTs with a high risk of bias and high level of heterogeneity was also rated as low.

## 3 BACKGROUND: EPIDEMIOLOGY OF ANEMIA IN PREGNANCY

Anemia is a major public health issue that affects 32 million (36%) pregnant women globally.^1^ Every year, it contributes to more than 115 000 maternal deaths and 591 000 perinatal deaths worldwide.^2^ Low‐ and middle‐income countries (LMICs) are the most affected, with 48.7% of pregnant women in these countries having anemia, and rates in the range of 20%–40% in more than half the countries.^3^, ^4^ In contrast, the prevalence of anemia in pregnancy in high‐income countries (HICs) is 15%.^1^ By region, African women bear the highest burden of anemia in pregnancy, with a prevalence rate of 57.1%, followed by South‐East Asia (48.2%), while the lowest prevalence is found among pregnant women in America (24.1%).^5^

WHO, in its 2016 Global Nutrition Report, declared that nearly all countries were off course for its target of a 50% reduction of maternal anemia by 2025.^6^ We are currently in the target year of 2025 and the global prevalence of anemia in pregnancy (36%) remains more than twice the 15% target.^6^ In more than two decades, the global prevalence of anemia in pregnancy has reduced by only 7%: from 43% in 1995 to 36% in 2019,^1^, ^6^ representing a marginal 3% decline per decade. This calls for renewed global focus on optimal approaches for reducing the burden and complications of anemia in pregnancy. The best approach is multisectoral, involving all relevant stakeholders and sectors. This paper represents the FIGO Committee on Childbirth and Postpartum Hemorrhage's contribution in this direction.

## 4 DEFINITION AND DIAGNOSIS OF ANEMIA IN PREGNANCY

In 1968, a WHO study group on nutritional anemias defined anemia in pregnancy as a hemoglobin concentration of less than 11.0 g/dL,^7^, ^8^ and in 1989, the WHO guide on preventing and controlling iron deficiency anemia through primary health care classified anemia in pregnancy based on severity into mild (10.0–10.9 g/dL), moderate (7.0–9.9 g/dL), and severe anemia: (<7.0 g/dL).^8^, ^9^ These cutoffs were applied to all pregnant women, irrespective of trimester of pregnancy, and remained unchanged until 2024, when WHO reclassified anemia and its severity based on trimester of pregnancy. The updated guideline defines anemia as a hemoglobin concentration of less than 11.0 g/dL in the first and third trimesters and less than 10.5 g/dL in the second trimester.^10^ This change was premised on the fact that owing to hemodilution from an increase in maternal plasma volume during pregnancy, hemoglobin concentrations decline during the first trimester, reaching their nadir in the second trimester, and begin to rise again in the third trimester.^8^ Cutoffs for defining mild, moderate, and severe anemia in the first and third trimesters remain the same. However, the following classifications are applied in the second trimester: mild anemia (9.5–10.4 g/dL), moderate anemia (7.0–9.4 g/dL), and severe anemia (<7.0 g/dL) (10).

Like WHO, the American College of Obstetricians and Gynecologists (ACOG) and the Centers for Disease Control and Prevention (CDC) also define anemia as hemoglobin levels less than 11.0 g/dL in the first and third trimesters, and less than 10.5 g/dL in the second trimester.^11^, ^12^ The UK guidelines define anemia as hemoglobin concentration of less than 11/0 g/dL in the first trimester, less than 10.5 g/dL in the second and third trimesters, and less than 10.0 g/dL in the postpartum period.^13^ In some African settings, a lower hemoglobin cutoff of less than 10.0 g/dL is used to define anemia in pregnancy,^14^, ^15^, ^16^ owing to the fact that African populations generally have lower hemoglobin levels than white individuals.^17^ FIGO recommends that anemia is defined as hemoglobin concentration of less than 11.0 g/dL globally for all populations of women in all trimesters of pregnancy and during the postpartum period.^18^ This is to ensure uniformity in interpretation and comparison of research data from different parts of the world.

## 5 CAUSES OF ANEMIA IN PREGNANCY

The causes of anemia in pregnancy include nutritional deficiencies of iron, folic acid, vitamins A and B12, hemoglobinopathies such as sickle cell disease and thalassemia, obstetric hemorrhage, chronic infections like HIV and tuberculosis (TB), hemolytic anemia, aplastic anemia, and in low‐income countries, parasitic infestations such as malaria, schistosomiasis, and hookworm. This paper discusses nutritional deficiencies, hemoglobinopathies, and parasitic infestations.

## 6 IRON DEFICIENCY ANEMIA

### 6.1 Iron requirements and absorption in pregnancy

Iron deficiency is the most common cause of anemia in pregnancy, being responsible for 50% of all cases of anemia in pregnant women worldwide.^19^ Owing to an increase in maternal red blood cell mass, placental and fetal growth, and blood loss at birth, iron requirements increase 10‐fold during pregnancy, with total requirements approximating 1200 mg.^20^, ^21^ This translates to an average daily demand for iron in pregnancy of approximately 4.4 mg.^22^ Daily requirements increase from 0.8 mg/day in the first trimester to 4–5 mg/day in the second trimester and furthermore to 7.5 mg/day in the third trimester, reaching up to 10 mg/day in the last 6–8 weeks of pregnancy.^20^, ^21^ Iron demands are provided by two major dietary sources, namely heme iron and non‐heme iron. Heme iron is ferrous iron (Fe2+) contained in animal food sources such as meat, fish, and poultry, while non‐heme iron is ferric iron (Fe3+) present in plant foods.^19^ Iron is absorbed in the ferrous form, whereas ferric iron needs to first be broken down to the ferrous form before absorption. Thus, the rate of absorption of heme iron (15%–35%) is higher than the absorption of non‐heme iron (1%–15%).^19^, ^23^ A typical diet provides 15 mg of elemental iron per day, of which only approximately 10% is absorbed.^11^, ^24^ The rate of absorption increases up to 30% during pregnancy, providing up to 5 mg of elemental iron per day.^21^, ^25^ Gastric acid, vitamin C, retinol, and carotenes enhance iron absorption, while phytates in cereals, calcium, antacids, tannins in tea and coffee, soy protein, milk, egg, and red wine taken together with iron containing food, inhibit its absorption.^13^, ^26^

### 6.2 Iron supplementation in pregnancy

Even with optimal diet and absorption, daily iron requirements in pregnancy cannot be met by diet alone, hence the need for iron supplementation.^21^ This is more so as up to 40% of pregnant women start pregnancy with depleted iron stores.^22^ Available evidence supports routine iron supplementation in pregnancy, to reduce the incidence of maternal iron deficiency and iron deficiency anemia at term, and small for gestational age (SGA) and low birth weight babies.^27^, ^28^, ^29^ All pregnant women should receive daily iron supplementation with 30–60 mg of elemental iron.^12^, ^18^, ^30^ A higher daily dose of 60 mg is recommended in settings where iron deficiency anemia is a significant public health problem affecting 40% or more of pregnant women, and in women at increased risk of iron deficiency, including those with previous anemia, multiple pregnancy, short interpregnancy interval of less than 1 year, and vegetarians.^30^, ^31^, ^32^

Systematic and Cochrane reviews have demonstrated that intermittent oral iron supplementation with 120 mg of elemental iron/week produces similar maternal and infant outcomes as daily supplementation, with fewer side effects.^33^, ^34^ Intermittent supplementation is recommended if daily iron is not tolerated due to side effects and in settings where iron deficiency anemia affects less than 20% of pregnant women.^30^

### 6.3 Screening and diagnosis of iron deficiency anemia in pregnancy

All pregnant women should be screened for anemia, with a FBC at booking and again at 28 weeks.^13^, ^18^ Screening can also be conducted with a hemoglobin concentration or hematocrit test, especially in resource‐limited settings.^12^, ^30^ Women with a hemoglobin concentration below 11.0 g/dL should be evaluated for the etiology of anemia. A serum ferritin level below 30 μg/L is diagnostic of iron deficiency anemia.^35^, ^36^ In the absence of a serum ferritin test, especially in resource‐limited settings, a positive response to a therapeutic trial of oral iron, as evidenced by an increase in hemoglobin concentration of at least 1 g/dL after 2 weeks of commencement of treatment is diagnostic of iron deficiency anemia.^13^, ^36^ In settings where extensive investigations to determine the cause of anemia may be difficult—in the absence of any concurrent/background illnesses/comorbidities/hemoglobinopathy—iron deficiency anemia can be presumed if the hemoglobin concentration is below 11 g/dL, and treatment with iron should be offered as first‐line therapy.^12^

### 6.4 Treatment of iron deficiency anemia in pregnancy

**FIGURE 1:**
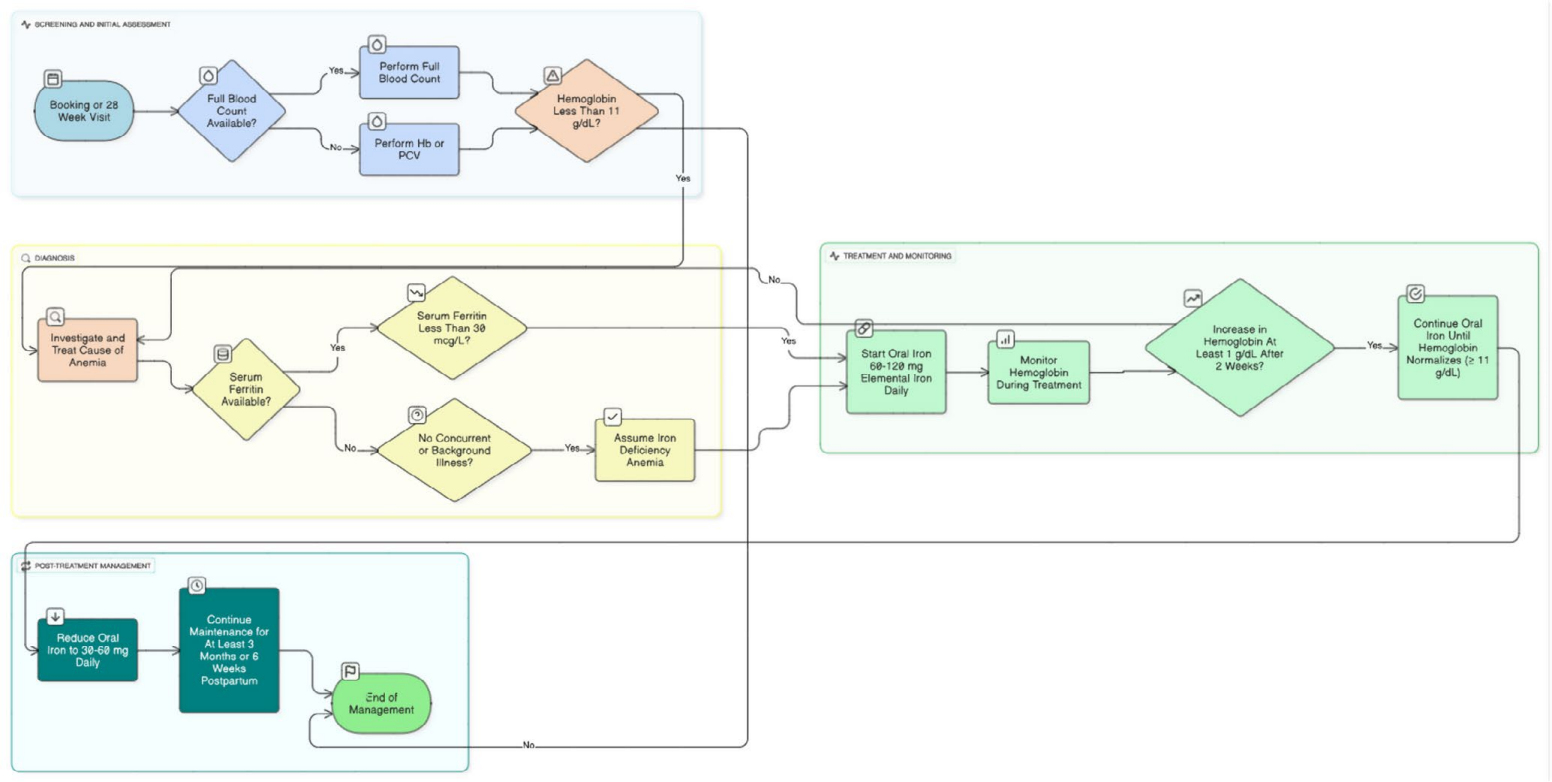
Algorithm for the screening, diagnosis, and treatment of iron deficiency anemia in pregnancy.

Treatment of iron deficiency anemia with oral iron as first line, at a dose of 60–120 mg of elemental iron per day, is recommended (Figure 1).^12^, ^30^, ^32^ FIGO previously recommended a treatment dose of 100–200 mg;^18^ however, it has been demonstrated that plasma hepcidin concentration increases rapidly after iron administration, remaining increased for up to 48 h, and negatively influencing absorption of subsequent iron doses. Iron doses of more than 60 mg amplify hepcidin increase and decrease fractional iron absorption.^37^ Furthermore, lower doses of iron have been demonstrated to be as effective as higher doses for the treatment of iron deficiency anemia, with fewer side effects.^38^ For these reasons, lower dosages of iron (40–80 mg) given as single daily doses (as against divided doses) on alternate days (as against consecutive days) have been suggested to improve fractional iron absorption, as well as tolerability and compliance by reducing gastrointestinal side effects.^37^, ^39^, ^40^ However, a recent systematic review by Kamath et al.^41^ demonstrated no significant difference in hemoglobin levels between daily and alternate day oral iron administration, even though gastrointestinal side effects were reduced with alternate‐day administration.

Treatment for iron deficiency anemia should be continued until hemoglobin concentration normalizes at 11.0 g/dL or above. Thereafter, the dose of iron should be reduced to the prophylactic dose of 30–60 mg of elemental iron per day to prevent a recurrence of anemia (Figure 1).^12^, ^30^ This should be continued for at least 3 months or 6 weeks postpartum, whichever is longer, to replenish iron stores (Figure 1).^13^, ^18^, ^36^ To maximize absorption, oral iron is best taken in the morning on an empty stomach (1 h before eating or 2 h after), and 1 h before or 2 h after inhibitors of iron absorption, such as calcium, proton pump inhibitors, antacids, thyroxine, tea, coffee, milk, soy, and eggs.^42^, ^43^, ^44^ Evidence supporting vitamin C administration to improve iron absorption is conflicting.^44^, ^45^, ^46^, ^47^, ^48^ Further studies are needed to make definite recommendations.

Parenteral iron is indicated after the first trimester for the treatment of iron deficiency anemia in women who do not respond to oral iron (less than 1 g/dL increase in hemoglobin concentration after 2 weeks of treatment with oral iron or less than 2 g/dL after 4 weeks), intolerance to oral iron, poor compliance with oral iron, moderate to severe iron deficiency anemia, iron deficiency anemia occurring within 4–6 weeks of anticipated childbirth, and women with a history of gastric surgery or conditions like inflammatory bowel disease that impair absorption of oral iron (Table 1).^35^, ^42^, ^49^ Parenteral iron is not advised in the first trimester owing to a lack of safety data.^36^ Blood transfusion is indicated in women with severe anemia, especially when it is close to anticipated time of childbirth, acute severe bleeding or where there is a risk of further bleeding, and for significant symptomatic anemia with features of hemodynamic or cardiac compromise (Table 1).^18^, ^36^

**TABLE 1:** Indications for parenteral iron and blood transfusion in iron deficiency anemia in pregnancy.

Intervention	Indications
Parenteral iron	Poor/no response to oral iron (<1 g/dL increase in hemoglobin concentration after 2 weeks of treatment with oral iron or <2 g/dL after 4 weeks)
	Intolerance to oral iron
	Poor compliance with oral iron
	Moderate to severe iron deficiency anemia occurring within 4–6 weeks of anticipated childbirth
	History of gastric surgery or conditions like inflammatory bowel disease that impair absorption of oral iron
Blood transfusion	Severe anemia close to anticipated time of childbirth
	Acute severe bleeding or where there is a risk of further bleeding
	Significant symptomatic anemia with features of hemodynamic or cardiac compromise

## 7 FOLATE DEFICIENCY IN PREGNANCY

Folate is the naturally occurring form of vitamin B9, while folic acid is its synthetic form contained in fortified foods and dietary supplements.^50^ Folate is abundant in fruits, meat, liver, and leafy vegetables: the term folate is derived from the Latin word “folium,” which means leaf, as it was extracted from spinach leaves by Mitchell et al.^51^ in 1941. The body stores 10–20 mg of folate in the liver, which is sufficient for only 2–3 months without dietary supplementation, with megaloblastic anemia occurring after about 4 months of eating a folate‐deficient diet.^52^ Folate is heat labile and destroyed when food is over‐cooked; therefore, folate deficiency can occur rapidly.^52^ In addition, whereas synthetic folic acid is 100% bioavailable when taken on an empty stomach (the bioavailability when taken with food is approximately 85%), the bioavailability of food folate is 50% or less.^53^ These highlight the need for folic acid supplementation in pregnancy to prevent folate deficiency. Folate deficiency causes macrocytic or megaloblastic anemia, fetal neural tube defects, fetal growth restriction, preterm birth, and low birth weight.^51^, ^54^ Causes of folate deficiency include inadequate dietary intake, malabsorption, use of anti‐folate drugs, and increased demands, as in pregnancy.^53^ Whereas folate deficiency is rare (0%–5%) in HICs, it is not uncommon (40%–50%) in LMICs.^55^, ^56^, ^57^

### 7.1 Folic acid supplementation in pregnancy and pre‐pregnancy

The recommended dietary intake (RDI) for folate increases from 180 to 190 μg/day in the normal non‐pregnant female adult to 500–600 μg/day during pregnancy.^58^, ^59^, ^60^ It has been demonstrated that 300 μg/day of synthetic folic acid from supplements and fortified foods plus 100 μg/day of dietary folate is sufficient to meet the increased folate demands during pregnancy.^53^, ^60^ For this reason, daily oral folic acid supplementation with 400 μg (0.4 mg) of folic acid, given together with oral iron, is recommended to prevent maternal anemia and other complications of folate deficiency in pregnancy.^30^ Pregnant women should also be counseled to eat folate‐rich and folate fortified foods.

To prevent fetal neural tube defects, it is recommended that all women of reproductive age who are planning to get pregnant should commence folic acid at a dose of 400 μg/day at least 2–3 months before pregnancy and continue until 12 weeks of pregnancy.^61^, ^62^, ^63^ A high daily dose of folic acid of 5 mg commenced at least 2–3 months before conception and continued until 12 weeks of pregnancy is recommended for the prevention of fetal neural tube defects in the following categories of women at high risk of fetal neural tube defects: women (or their partners) who have neural tube defect or a family history of neural tube defect or other congenital malformations; women with a previous pregnancy that was affected by neural tube defect or other congenital malformations; women with type 1 or 2 diabetes mellitus; women with obesity with a BMI of 30 or above; and women on anti‐folate medications.^61^, ^62^, ^63^ After 12 weeks of pregnancy, the dose can be reduced to 400 μg/day and taken (together with iron) throughout pregnancy to prevent maternal anemia and other complications of folate deficiency in pregnancy aside from fetal neural tube defects. However, women with hemoglobinopathies, women on anti‐folate drugs, and those with a BMI of 30 or above should continue folic acid at the high dose of 5 mg/day throughout pregnancy. It has been demonstrated that women with obesity have lower folate levels and higher odds of folate deficiency than their normal‐weight counterparts due to several factors, including chronic low‐grade inflammation that increases metabolic demands, insulin resistance, poor quality‐diet adherence, and poor compliance with folic acid supplementation.^55^, ^64^, ^65^, ^66^, ^67^

A Cochrane review by Peña‐Rosas et al.,^33^ and more recently a systematic review and meta‐analysis by Chillo et al.,^68^ showed that intermittent supplementation with 2800 μg (2.8 mg) of folic acid/week (together with iron) is as effective as daily supplementation for the prevention of maternal anemia, with similar maternal and neonatal outcomes, fewer gastric side effects, and a higher likelihood of compliance. Intermittent folic acid supplementation should be considered in women who do not tolerate the gastric side effects of daily oral iron/folic acid and in settings where anemia affects less than 20% of pregnant women.^30^ Intermittent folic acid supplementation has been also demonstrated to be effective for the prevention of fetal neural tube effects.^69^ However, it is not recommended in women with a BMI of 30 or above and women on anti‐folate drugs, as these women require higher doses of folic acid.

## 8 VITAMIN B12 DEFICIENCY IN PREGNANCY

Vitamin B12 (cobalamin) is a cobalt‐containing, water‐soluble vitamin that is found predominantly in animal sources including meat, fish, eggs, milk, cheese, and artificially fortified food.^52^, ^70^ It acts as a co‐enzyme in the methylation of homocysteine to methionine, a rate‐limiting step in the conversion of folate to metabolically active forms that are required as co‐enzymes for DNA synthesis. In vitamin B12 deficiency, active forms of folate are not formed and DNA synthesis is consequently impaired, resulting in megaloblastic anemia.^52^ The principal causes of vitamin B12 deficiency are low dietary intake, as in vegetarian diets, and malabsorption disorders, usually due to pernicious anemia.^70^

### 8.1 Vitamin B12 supplementation in pregnancy

Some studies have suggested possible benefits of vitamin B12 supplementation in pregnancy.^71^, ^72^ However, a recent Cochrane review of five trials including 984 women by Finkelstein et al.^73^ found that although vitamin B12 supplementation may reduce the risks of maternal vitamin B12 deficiency and maternal anemia, and improve maternal and infant vitamin B12 concentrations, evidence to support these findings were uncertain. There is also uncertainty about the effects of vitamin B12 supplementation on other maternal and neonatal outcomes, such as miscarriage, neural tube defects, preterm birth, and low birth weight.^73^, ^74^ Therefore, there is currently no strong evidence to recommend routine vitamin B12 supplementation in pregnancy to prevent megaloblastic anemia. In addition, while maternal stores of vitamin B12 (mainly in the liver and kidney) average 3–4 mg, the RDI for vitamin B12 in pregnancy is 2.6 μg/day (just 0.2 μg/day higher than the RDI for non‐pregnant women) and the total fetal vitamin B12 requirement in pregnancy is 50 μg.^52^, ^70^ Maternal stores of vitamin B12 are sufficient to meet vitamin B12 demands for approximately 3 years without dietary intake; therefore, vitamin B12 deficiency is very rare, occurring in less than 0.1% of young adults and 1%–2% of elderly individuals.^52^ Furthermore, folate supplementation may mask clinical symptoms of vitamin B12 deficiency by exogenously replacing folate that is inactivated in vitamin B12 deficiency.

## 9 MULTIPLE MICRONUTRIENT DEFICIENCY IN PREGNANCY

The most common and studied micronutrient deficiencies include iron, folate, vitamin A, iodine, and zinc, although many other micronutrient deficiencies exist. Coexistence of multiple micronutrient deficiencies is common, affecting more women in LMICs and low‐resource settings.^50^ A national micronutrient survey in Bangladesh revealed that 28.9% of non‐pregnant and non‐lactating women had anemia, with deficiencies of vitamin D (69.9%), zinc (43.3%), iodine (26.9%), folic acid (29.0%), vitamin B12 (20.2%), iron (14.1%), and vitamin A (7.5%), justifying multiple micronutrient supplementation in pregnancy.^75^ A systematic review of 52 studies on micronutrient intakes of women in resource‐poor settings reported that, except for vitamin A (29%), vitamin C (34%), and niacin (34%), the reported mean/median intakes in over 50% of studies were below the estimated average requirements.^76^

### 9.1 Multiple micronutrient supplementation in pregnancy

Systematic and Cochrane reviews have demonstrated maternal and neonatal benefits of multiple micronutrient supplementation (containing iron and folic acid) during pregnancy, including a reduction in preterm births, small for gestational age, and low birth weight.^77^, ^78^, ^79^, ^80^, ^81^, ^82^ The 2016 WHO antenatal care guidelines noted that there is some evidence of additional benefits of multiple micronutrient supplements over iron and folic acid supplements alone during pregnancy but also suggested some risks and gaps in evidence.^30^ However, systematic and Cochrane reviews conducted after the WHO antenatal care guidelines of 2016 did not report any maternal or neonatal risks.^79^, ^80^, ^81^, ^82^ There is a growing consensus that multiple micronutrient supplements (containing iron and folic acid) should be considered over iron and folic acid supplements alone for pregnant women in LMICs and in populations with a high prevalence of nutritional deficiencies among women of reproductive age.^30^, ^80^, ^81^ It is recommended that multiple micronutrient supplements used in pregnancy should contain the following 15 micronutrients at doses that meet the recommended dietary allowances for these micronutrients: folic acid (400 μg), iron (30 mg), copper (2 mg), iodine (150 μg), selenium (65 μg), vitamin A (800 μg), vitamin B1 (thiamin, 1.4 mg), vitamin B2 (riboflavin, 1.4 mg), vitamin B3 (niacin, 18 mg), vitamin B6 (pyridoxine, 1.9 mg), vitamin B12 (cobalamin, 2.6 μg), vitamin C (ascorbic acid, 70 mg), vitamin D (200 IU), vitamin E (10 mg), and zinc (15 mg).^83^ Multiple micronutrient supplements should be administered once daily throughout pregnancy. To prevent fetal neural tube defects, they should be commenced at least 2–3 months before pregnancy.^62^, ^83^ Intermittent/weekly administration of multiple micronutrient supplements in pregnancy is not recommended.^83^

Multiple micronutrient supplements containing 5 mg of folic acid should be started 2–3 months before pregnancy, given once daily, in women at high risk of fetal neural tube defects and continued until 12 weeks gestation, and thereafter changed to multiple micronutrient supplements containing 400 μg of folic acid, which should be continued once daily throughout pregnancy,^62^, ^84^ except for women with obesity with a BMI of 30 or above and women on antifolate drugs, who should maintain a high daily dose of 5 mg of folic acid throughout pregnancy, as already discussed above. In the absence of a multiple micronutrient supplement containing 5 mg of folic acid, additional tablet(s) of folic acid should be taken in addition to the multiple micronutrient supplement to achieve a daily dose of 5 mg of folic acid.^83^, ^84^

It is not recommended for pregnant women to use multiple micronutrient supplements containing more than 30 mg of iron owing to side effects.^83^ For women at high risk of iron deficiency anemia, in populations where iron deficiency anemia is a significant public health problem affecting 40% or more of pregnant women, and for the treatment of iron deficiency anemia, where 60–120 mg of iron is required, rather than use a multiple micronutrient supplement containing such high dose of iron, additional iron tablet(s) should be administered in addition to a multiple micronutrient supplement containing 30 mg of iron to achieve the desired daily iron dose.^83^

## 10 HEMOGLOBINOPATHY IN PREGNANCY

Approximately 1.5 million pregnancies worldwide are at risk of hemoglobinopathies annually, with 9.2 million pregnant carriers and 365 100 infants affected by hemoglobinopathies every year.^85^ All pregnant women should be screened with a FBC and hemoglobin electrophoresis at booking to detect hemoglobinopathies in pregnancy.^86^

### 10.1 Folic acid supplementation in pregnant women with hemoglobinopathy

Women with hemoglobinopathy require increased folic acid supplementation with 5 mg/day of folic acid before and during pregnancy owing to the continual turnover of red blood cells, to reduce the risk of fetal neural tube defects and compensate for increased demand in pregnancy.^87^, ^88^, ^89^ Folic acid supplementation can be provided with either a 5 mg folic acid tablet or a multiple micronutrient supplement containing 5 mg of folic acid. In the absence of a multiple micronutrient supplement containing 5 mg of folic acid, additional folic acid tablet(s) should be given in addition to the multiple micronutrient supplement to achieve a daily dose of 5 mg of folic acid.^83^, ^84^ Multiple micronutrient supplementation is beneficial because the high metabolic expenditure and increased demands in pregnancy put pregnant women with sickle cell disease at high risk of malnutrition and micronutrient deficiencies.^90^, ^91^ Intermittent folic acid supplementation is not recommended in women with sickle cell disease as these women require a higher daily dose of 5 mg folic acid compared to the 2.8 mg weekly dose in intermittent supplementation.

### 10.2 Iron supplementation in pregnant women with hemoglobinopathy

Routine iron supplementation in pregnant women with hemoglobinopathy is not recommended, because of the risk of iron overload from repeated blood transfusions.^89^ In addition to an FBC at booking, pregnant women with known hemoglobinopathy should undergo a serum ferritin test and only commenced on iron supplementation if the level of ferritin is below 30 μg/L (i.e. confirmed iron deficiency).^13^, ^87^ However, in settings where serum ferritin testing is not readily available, iron supplementation can be safely administered to pregnant women with sickle cell disease who have no history of repeated blood transfusions. This is due to the fact that in contrast to other hemolytic anemias, hemolysis in sickle cell disease is associated with iron deficiency; in the absence of repeated blood transfusions, stored iron rarely exceeds 2000 mg in sickle cell disease.^92^ This is because whereas the hemolysis in thalassemia major is intra bone marrow, it is predominantly intravascular in sickle cell disease. Bone marrow‐derived factors suppress hepcidin production in thalassemia, resulting in increased dietary absorption of iron, which is not the case in sickle cell disease.^92^, ^93^ Subsequent urinary and biliary iron elimination after intravascular hemolysis, as well as increased dietary requirement in pregnancy, produces iron deficiency in sickle cell disease.^92^, ^94^, ^95^

Studies, including a systematic review by Aroke et al., have shown that iron deficiency is prevalent in pregnant women with sickle cell disease, and iron supplements can be given to these women if they have no history of repeated blood transfusions.^96^, ^97^, ^98^, ^99^, ^100^ Iron supplementation at a dose of 30 mg of elemental iron per day can be provided with either iron tablet or a multiple micronutrient supplement containing 30 mg of iron. Women who do not tolerate daily iron owing to side effects can have intermittent supplementation with 120 mg of iron/week.^30^, ^33^, ^34^

### 10.3 Blood transfusion in pregnant women with hemoglobinopathy

Blood transfusion is indicated to treat acute anemia in pregnant women with sickle cell disease, but there is no consensus on the exact hemoglobin concentration below which transfusion should be considered. Most authors recommended transfusion when the hemoglobin concentration is below 5–7 g/dL (the steady‐state hemoglobin concentration in sickle cell disease is usually in the range of 6–8 g/dL), or above 2 g/dL below the steady‐state hemoglobin concentration, or above 20% reduction from the steady‐state hemoglobin concentration.^101^, ^102^, ^103^, ^104^

Current evidence from systematic and Cochrane reviews does not support routine prophylactic blood transfusion in pregnant women with sickle cell disease.^105^, ^106^ Restricting blood transfusion in pregnant women with sickle cell disease to situations in which it is clinically indicated reduces the risks of alloimmunization, viral infections, and iron overload.^86^ For pregnant women with thalassemia syndromes, hemoglobin concentration should be maintained at a target of 10 g/dL with transfusions to avoid maternal and fetal morbidity.^86^, ^88^

## 11 MALARIA IN PREGNANCY

Globally, 125 million pregnant women reside in areas where they are at risk of contracting malaria in pregnancy.^107^ In Africa, which has the highest global prevalence of malaria in pregnancy, WHO reported that of the 40 million pregnancies that occurred in the region in 2021, 13.3 million were affected by malaria.^108^ In sub‐Saharan Africa (SSA), 26% of severe anemia in pregnancy is attributed to malaria, with malaria‐induced anemia estimated to contribute to nine maternal deaths per 100 000 live births in endemic areas.^109^ Prevention of malaria in pregnancy reduces maternal severe anemia by 38%.^109^

### 11.1 Malaria chemoprophylaxis in pregnancy

For the prevention of malaria in pregnancy, in addition to the use of long‐lasting insecticidal nets, WHO recommends intermittent preventive treatment with IPT_P_‐SP for all pregnant women in malaria‐endemic areas, beginning as early as possible in the second trimester, with subsequent doses given at least 4 weeks apart until birth, ensuring at least three doses before birth.^30^, ^110^ IPT_P_‐SP should be administered as DOT in the antenatal clinic with 1500 mg of sulfadoxine/75 mg of pyrimethamine (three tablets of SP, each containing 500 mg of sulfadoxine/25 mg of pyrimethamine).^110^

SP acts by inhibiting folate synthesis and co‐administration with high‐dose folic acid (≥5 mg) has been found to counteract its efficacy and is, thus, not recommended.^30^, ^89^, ^110^, ^111^, ^112^, ^113^, ^114^ Low‐dose folic acid (0.4–1.5 mg) has no effect, and only this dose should be co‐administered with SP.^30^, ^89^, ^110^, ^111^, ^112^, ^113^, ^114^, ^115^ However, in most African countries, only 5 mg tablets of folic acid are available and used by all pregnant women.^112^, ^113^ FIGO supports the advocacy by WHO that these countries should prioritize the procurement and distribution of low‐dose folic acid (400 μg) for use by pregnant women, and high‐dose folic acid (5 mg) should only be used by specific categories of pregnant women: those at high risk of fetal neural tube defects and those with hemoglobinopathies.^116^

For women at high risk of fetal neural tube defects, it is recommended that high dose (5 mg) folic acid be switched to low dose (400 μg) at 12 weeks of pregnancy,^61^, ^62^ after which IPT_P_‐SP is commenced. For women with a BMI of 30 or above, women on antifolate drugs and women with hemoglobinopathies, who require high‐dose (5 mg) folic acid throughout pregnancy, switching to low‐dose (0.4–1.5 mg) folic acid for 14 days after administration of SP for IPT should be considered based on the available evidence.^111^, ^114^

SP is contraindicated before 13 weeks of pregnancy owing to teratogenic concerns; in women taking other sulfa‐containing drugs for prophylaxis such as co‐trimoxazole in HIV, to avoid sulfa overdose and adverse effects; and in women who are allergic to any of the components of SP.^110^, ^116^ For women who are allergic to any of the components of SP, there is limited research and evidence on the efficacy of alternative drugs to SP for IPT.^110^, ^117^ More research is needed in this area, especially with growing *Plasmodium falciparum* resistance to SP.^118^ Pregnant women who are allergic to any of the components of SP should be encouraged to use long‐lasting insecticidal nets and adopt other malaria‐prevention strategies, with early diagnosis and prompt treatment of clinical malaria.

### 11.2 Malaria chemoprophylaxis in pregnant women with hemoglobinopathy

Pregnant women with sickle cell disease are particularly susceptible to the lethal effects of malaria. Malaria worsens the anemia of sickle cell disease, can trigger pain or sequestration crisis in affected women, and the hypo−/asplenism often seen in association with sickle cell disease reduces the clearance of parasitized red blood cells.^119^ For these reasons, routine malaria chemoprophylaxis in sickle cell disease is beneficial and recommended in malaria‐endemic areas.^120^, ^121^ Malaria chemoprophylaxis with daily proguanil is recommended because proguanil resistance is less widespread, with good pharmacologic and safety profile.^122^, ^123^, ^124^ This is also the recommendation in Nigeria,^124^ which currently has the highest burden of both malaria and sickle cell disease globally.^125^, ^126^

## 12 HELMINTHIC INFESTATIONS AND ANTI‐HELMINTHIC PROPHYLAXIS IN PREGNANCY

Helminthic infestations are among the major public health issues in tropical and resource‐limited countries. Approximately 44 million pregnant women are affected globally, with 6.9–7.5 million of them residing in SSA; this translates to between one third to one fourth of pregnancies in SSA.^127^, ^128^ Helminthic infestations contribute to severe anemia through blood loss, micronutrient deficiencies, and malnutrition.

A Cochrane review of four trials including 4265 participants found some evidence of a beneficial effect of anthelminthic treatment in pregnancy on maternal anemia in one of the studies.^129^ Based on this, WHO recommends preventive chemotherapy in pregnancy with a single‐dose of 400 mg albendazole or 500 mg mebendazole administered after the first trimester to all pregnant women residing in areas where both the prevalence of helminthiasis is 20% and above and anemia is a severe public health problem affecting 40% or more of pregnant women.^30^, ^130^

## 13 ANEMIA AND PPH

Maternal anemia is a significant risk factor for mortality and morbidity from PPH.^131^ Anemia reduces the oxygen‐carrying capacity of the blood and in severely anemic pregnant women, as little as 250 mL of blood loss can cause a significant compromise in hemodynamic status, while healthy women can well tolerate up to 1000 mL of acute blood loss.^132^, ^133^

Anemia predisposes to PPH by one or a combination of several mechanisms. Blood flow from bleeding vessels is increased by a reduction in blood viscosity seen in association with a reduced hematocrit, combined with maternal tachycardia and increased cardiac output, both compensatory to maintaining oxygen delivery in anemia. Anemic blood clots are fragile and more susceptible to fibrinolysis, and uterine hypoxia from anemia impairs myometrial contractility resulting in uterine atony, which is the most common cause of PPH.^131^ Therefore, every effort must be made to prevent, screen for, and correct antepartum anemia before childbirth, especially in settings where pregnant women are at a higher risk of anemia. We recommend the adoption of a universal hemoglobin concentration cutoff of less than 11 g/dL for diagnosing anemia in all trimesters of pregnancy, rather than the cutoff of 10 g/dL currently in use in some African settings^14^, ^15^, ^16^ This is even more so as women in these settings are more prone to anemia due to prevalent nutritional deficiencies. Women who start pregnancy with a hemoglobin concentration of 11 g/dL or above will tolerate more postpartum blood loss, with a lower risk of PPH, than women with a hemoglobin concentration of 10 g/dL.

## 14 CONCLUSIONS

Anemia is a significant public health concern, affecting more than one third of pregnant women globally. Major causes include nutritional deficiencies, hemoglobinopathies, malaria, and helminthiasis. All pregnant women should be screened for anemia in pregnancy, and prompt treatment instituted if it is identified. Nutritional interventions and other preventive strategies should be adopted to reduce the risk of antepartum anemia and associated complications, including PPH.

These FIGO good practice recommendations for the screening, diagnosis, and prevention of anemia in pregnancy summarize the available evidence‐based guidance, which, if adopted globally, we expect will significantly reduce the excessive maternal and perinatal mortality and morbidity due to anemia in pregnancy and associated complications, including PPH.

## AUTHOR CONTRIBUTIONS

All the authors contributed to the study conception and design. AEU carried out the literature search, synthesis of evidence, grading of recommendations and evidence, and wrote the first manuscript draft. FB, DR, AJN, MO, ZJN, JB‐K, and AW critically reviewed and revised the first manuscript draft for sound intellectual and current best evidence content. AW coordinated the reviews and revisions, which were implemented by AEU, to produce a second manuscript draft. The second manuscript draft was again critically reviewed and revised by all the authors to produce a third manuscript draft, which IN reviewed, revised, and edited to produce a final manuscript draft. All the authors read and approved the final manuscript draft.

## FUNDING INFORMATION

The authors received no funding for this study.

## CONFLICT OF INTEREST STATEMENT

The authors have no conflicts of interest.

## Data Availability

Data sharing is not applicable to this article as no new data were created or analyzed in this study.
